# IABP 9th General Assembly

**Published:** 2012

The 9th General Assembly (9GA) of the International Agency for the Prevention of Blindness (IAPB) was a resounding success, with over 1,400 people attending the five-day meeting in Hyderabad, India.

The assembly was superbly organised and was characterised by a spirit of learning and collaboration, not only during presentations and panel discussions, but also during chance meetings in corridors and around the tea and coffee tables.

We asked a few leaders in international non-governmental organisations what key messages they took home with them.

**Bob McMullan, incoming President of IAPB**: “We need to be very disciplined and prioritise because funds are limited. Working in a noble cause increases the obligation to be efficient and effective.”

**Serge Resnikoff, Organisation for the Prevention of Blindness**: “WHO is leading the movement towards universal health coverage; it will be up to all of us to ensure that, in our country, eye care is on the agenda when this is being discussed.”

**Lesley Podesta, Fred Hollows Foundation.** “There are so many unheralded women making a real difference in some of the most difficult environments and communities. We need to actively make efforts to bring more women into leadership roles over the next few years.”

**Allen Foster, CBM.** “Avoidable blindness is highest in neglected communities. In order to achieve the goal of VISION 2020 we need to focus our time, effort, and limited resources on improving eye care for these neglected communities.”

**Brien Holden, Brien Holden Vision Institute**: ‘We have to get down to the business of blindness prevention. We must become much more effective at bringing all the stakeholders together to focus on eliminating the problem. The corporate social responsibility panel at the 9GA showed that this is possible.”

**Figure:**
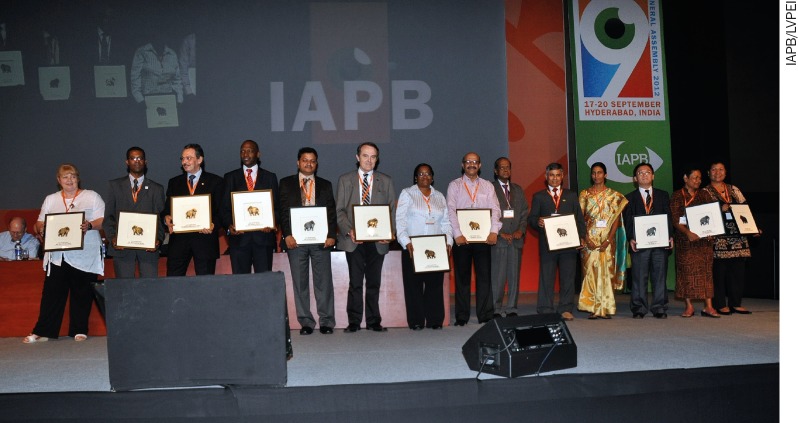
Recipients of IAPB 9th General Assembly awards

**Richard le Mesurier, IAPB Western Pacific**: “There needs to be a more pro-active focus on primary eye care. There are also various areas of neglect: Francophone Africa is lagging behind, and more work is needed to address refractive error and get spectacles to the poor.”

For more information about the general assembly, including presentations, videos and photographs, please visit: www.iapb.org/9th-general-assembly

